# Histoplasmosis or Blastomycosis in a Patient With Left Ventricular Assist Device

**DOI:** 10.7759/cureus.113048

**Published:** 2026-07-20

**Authors:** Kiran Gajurel

**Affiliations:** 1 Infectious Diseases, Carolinas Medical Center, Advocate Health, Charlotte, USA

**Keywords:** antigen, blastomyces, histoplasma, left ventricular assist device, sarcoidosis

## Abstract

Histoplasma and Blastomyces are environmentally acquired fungi that can cause a wide spectrum of clinical disease, ranging from asymptomatic to life-threatening illness. These fungal infections are rarely encountered in patients with a left ventricular assist device (LVAD), with only one case of histoplasmosis reported in the literature. We report histoplasmosis or blastomycosis with possible infection of the underlying LVAD. The patient is a 55-year-old male with an implantable cardioverter-defibrillator (ICD) and an LVAD for sarcoid-related cardiomyopathy. He was on infliximab for sarcoidosis and presented with a fever of unclear etiology along with fatigue, poor appetite, and weight loss. Blood and urine cultures were negative. There was no drainage of the drive line (DL) exit site. A CT of the chest and abdomen showed nonspecific pulmonary ground-glass opacities and no intra-abdominal abscess. Eventually, urine Histoplasma and serum Blastomyces antigens returned positive. The patient was started on itraconazole, and infliximab was stopped. The patient started improving, and Histoplasma and Blastomyces antigen levels started declining. Since the fungal blood culture was negative and there was no end-organ disease for biopsy or fungal culture, we could not distinguish between histoplasmosis and blastomycosis. This case underscores the rarity of histoplasmosis/blastomycosis in patients with LVADs.

## Introduction

Histoplasma and Blastomyces are thermally dimorphic fungi that exist as molds in the environment and as yeasts in the human host. They are usually acquired via inhalation of environmental fungal conidia and rarely via traumatic skin inoculation, organ transplantation, and reactivation of dormant infection. Although most infections are asymptomatic in immunocompetent patients, severe life-threatening infections (e.g., pulmonary and disseminated) can occur, especially in the immunocompromised population. In the United States, these infections were historically confined to the central and eastern states. In recent years, the incidence of these fungal infections has increased and expanded to areas outside traditionally designated endemic areas [1]. Although these fungi are known to cause infection of cardiac devices, infection in patients with a left ventricular assist device (LVAD), which is used in people with end-stage heart failure, is uncommon. Using search terms "Histoplasma," "histoplasmosis," "Blastomyces," and “blastomycosis” combined with “left ventricular assist device" or “LVAD” in PubMed, limited to the English language through June 30, 2026, we found only one published report of histoplasmosis and none of blastomycosis in patients with LVAD [2]. Here, we present a case of histoplasmosis or blastomycosis, highlighting their rarity in patients with LVAD.

## Case presentation

A 55-year-old man with a history of sarcoidosis involving the lungs, liver, and heart underwent an implantable cardioverter-defibrillator (ICD) and an LVAD implantation for sarcoid-related cardiomyopathy nine years and 21 months, respectively, before hospital admission. He had been on infliximab for about 10 months. Eleven months prior to admission, he developed methicillin-susceptible Staphylococcus aureus (MSSA) bacteremia without a clear source, raising a concern of underlying cardiac device infection. He was treated with cefazolin for five weeks after a negative blood culture, followed by long-term suppressive oral cephalexin. 

**Figure 1:**
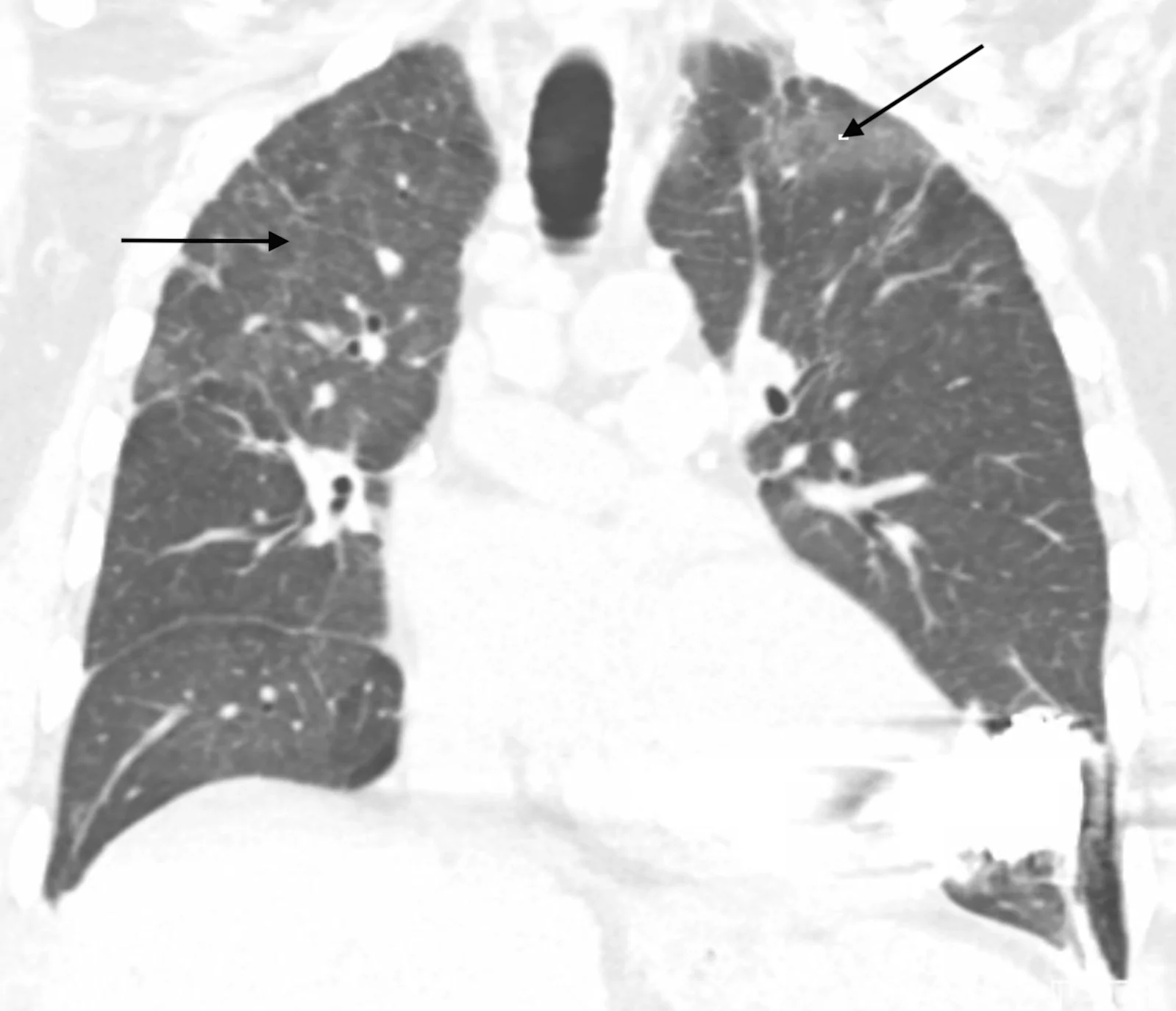
CT chest showing mild ground glass opacities (black arrows) in the upper lungs

On admission in November 2025, the patient endorsed fatigue, diarrhea, nausea, and emesis for about two weeks. He also had a poor appetite and a few pounds of weight loss. Diarrhea resolved spontaneously after admission. He did not feel feverish but was also noted to have a temperature up to 102.9 F without localizing signs and symptoms. There was no drainage from the drive line (DL) exit site. Non-contrast CT chest and abdomen showed vague mild ground-glass opacities in upper lobes with stable mediastinal lymphadenopathy (Figure 1). There was no intra-abdominal abscess. Blood and urine cultures were negative. He did not have cytopenia or peripheral eosinophilia. Since the patient looked stable otherwise, he was thought to have a drug fever, and cephalexin was switched to oral doxycycline 100 mg every 12 hours, and he was discharged home.

A few days after discharge, urine Histoplasma antigen (quantitative enzyme immunoassay) came back strongly positive at 19.5 ng/ml (reference < 0.2 ng/ml), and he was started on oral itraconazole (200 mg every eight hours for three days followed by 200 mg every 12 hours), and infliximab was stopped. Serum Blastomyces antigen (quantitative enzyme immunoassay) was also ordered before starting antifungal treatment, and it was also positive at 14.5 ng/ml (reference: none detected). The blood fungal culture was negative after four weeks of incubation. A month after initiating antifungal treatment, azathioprine was started for sarcoidosis. A PET CT around that time showed extensive cardiac scarring but with less activity; stable, metabolically active thoracic and mediastinal lymph nodes and stable multifocal regions of peribronchovascular thickening and nodularity in bilateral lungs compared to the PET CT done 18 months ago suggested stable sarcoidosis. A thoracic echocardiogram (TTE), done 12 weeks after initiation of treatment, did not show valvular vegetation. Follow-up urine Histoplasma and serum Blastomyces antigens started declining, and the patient felt better with the resolution of fever and fatigue. 

## Discussion

In the only published report of histoplasmosis in an LVAD patient, the fungal culture of the DL exit site drainage grew Histoplasma capsulatum [2]. There are no published reports of blastomycosis in LVAD patients. In the current report, blood fungal culture was negative, and there was no definitive tissue-invasive disease for biopsy or culture. The mediastinal and abdominal lymph nodes were active but stable (compared to prior imaging) and were attributed to sarcoidosis. The patient presented with nonspecific fatigue, loss of appetite, weight loss, and fever of unclear etiology, and the diagnosis of histoplasmosis or blastomycosis was based on elevated urine Histoplasma and serum Blastomyces antigen levels and a subsequent response to treatment with declining antigen levels. Unfortunately, we could not distinguish between histoplasmosis and blastomycosis due to significant cross-reactivity between their antigens [3, 4]. Histoplasma and Blastomyces antigens also cross-react with other fungi, especially with Paracoccidioidomyces and Talaromyces marneffei and, to a lesser extent, with Coccidioidomyces and Aspergillus species [3,5-7]. The patient, however, did not have epidemiologic risk factors for these fungal infections.

Histoplasma and Blastomyces are usually acquired via inhalation of environmental fungal conidia and rarely via traumatic skin inoculation, organ transplantation, and reactivation of dormant infection. The mode of acquisition in our patient is not clear. Since the patient did not have significant recent outdoor exposure, it is possible that this could have been a reactivation of a dormant infection. Blood fungal culture was not unexpectedly negative. Even in histoplasma endocarditis and solid organ transplants, histoplasmosis fungemia was seen in only 30% and 63% of patients, respectively (although fungemia in transplant patients correlates with the severity of disease) [8,9]. Fungemia is rare in blastomycosis [4].

Our patient had been on infliximab for sarcoidosis, and this likely predisposed him to the fungal infection. Tumor necrosis factor-alpha (TNF-a) inhibitors, especially infliximab, are a well-known risk factor for histoplasmosis [10-13]. Their association with blastomycosis is not as strong as in histoplasmosis [11]. However, it should be noted that both histoplasmosis and blastomycosis can occur in people with a normal immune system.

Unlike in the published report of histoplasmosis in an LVAD patient, where the DL infection was apparent, in our patient there was no clear involvement of the LVAD or the ICD. A CT chest/abdomen and PET CT did not suggest LVAD infection, and TTE did not show vegetation. It should be noted that a transesophageal echocardiogram, which is more sensitive than a TTE in detecting valve or lead vegetation, was not done. Similarly, a CT abdomen without contrast as well as a delay in obtaining a PET-CT (done a month after starting antifungals) might have missed a subtle infection of the LVAD. Moreover, imaging studies including PET-CT and leukocyte-labeled scintigraphy can have variable sensitivity and specificity in the diagnosis of LVAD infection [14,15]. However, due to the known association of these infections with cardiac devices, a possibility of underlying device infection, especially that of LVAD, cannot be excluded [9,16-20]. 

The management of Histoplasma/Blastomyces-related cardiac device infection can be challenging since definitive treatment may require device removal based on experience with Histoplasma-related prosthetic valve infection. In a review of 61 cases of Histoplasma endocarditis (36 with native valves and 25 with prosthetic valves), all patients who underwent both antifungal and surgical treatment survived, whereas the survival rate was only 50% among those who received antifungal treatment only [9]. However, LVAD removal or exchange is often not possible due to the morbidity associated with these procedures (unless they are candidates for heart transplantation). In a previous report of LVAD-associated histoplasmosis, the patient improved with antifungal treatment alone [2]. Since our patient clinically improved, the plan is currently to treat for at least one year and possibly indefinitely for suppression.

## Conclusions

Histoplasmosis and blastomycosis infections of the LVAD are unusual, but due to the increasing incidence of these fungal infections (even outside the historically designated endemic areas), a growing number of LVAD-related infections may be seen in the future. The presentation can be nonspecific. High diagnostic vigilance is required, especially in patients with negative blood and DL cultures, for prompt diagnosis of histoplasmosis and blastomycosis in patients with an LVAD.
